# The effect of family-based counseling on domestic violence in pregnant women referring to health centers in Sahneh city, Iran, 2018

**DOI:** 10.1186/s12991-021-00332-8

**Published:** 2021-02-05

**Authors:** Fereshteh Babaheidarian, Seyedeh Zahra Masoumi, Gita Sangestani, Godratollah Roshanaei

**Affiliations:** 1 Consultation in Midwifery, Department of Midwifery, Hamadan University of Medical Sciences, Hamadan, Iran; 2 Department of Midwifery, Mother and Child Care Research Center, School of Nursing and Midwifery, Hamadan University of Medical Sciences, Hamadan, Iran; 3 Modeling of Noncommunicable Diseases Research Center, Department of Biostatistics, School of Public Health, Hamadan University of Medical Sciences, Hamadan, Iran

**Keywords:** Counseling, Domestic violence, Pregnant women

## Abstract

**Background:**

Domestic violence is a term that shows aggressive behavior with different physical, psychological, economic, and social dimensions. This concept is considered as one of the health priorities regarding its significant effects on pregnancy, postpartum, and the outcome of pregnancy. The present study was conducted to investigate the effect of family-based counseling on domestic violence against pregnant women.

**Methods:**

An intervention trial was conducted on 92 pregnant women exposed to domestic violence, selected among 274 pregnant women accessible in Sahneh, Iran. Data collection was performed using the standard questionnaire of domestic violence against women of Mohseni Tabrizi. The pregnant women exposed to violence were randomly assigned to intervention and control groups (in two groups of 45 people). In the intervention group, three 45-min individual counseling sessions were held for the pregnant women and their spouses according to Greeting, Ask, Tell, Help, Explain, and Refer (GATHER) principles. Four weeks after the end of the sessions, the two groups again completed the questionnaire.

**Results:**

Family-based counseling intervention reduced the mean score of domestic violence in the intervention group from 68.58 ± 9.21 before the intervention to 49.56 ± 8.83 after intervention. Also, various domains of violence including mental, verbal, financial, physical, sexual, and social violence were significantly declined in the intervention group (*P* < 0.001); however, there was no significant decrease in emotional violence score (*P* > 0.001).

**Conclusion:**

Family-based counseling plays a significant role in reducing the various types of violence against women through increasing the awareness of couples and by improving the relationship between couples during pregnancy. It will reduce the incidence of violence against a pregnant woman and consequently reduce complications on the mother and pregnancy outcomes. Family-oriented counseling played a significant role in deterring all forms of violence against women by increasing awareness of couples and improving their relationship during pregnancy. Moreover, family-oriented counseling reduced the incidence of violence against pregnant women and thus deterred maternal complications associated with pregnancy.

## Introduction

Violence against women is one of the most important problems of human rights all over the world and is a major health, social, and public health problem. Besides, it is among the leading causes of death worldwide [1], which is spreading and involving more and more couples every day despite the advances in the humanities [2]. Violence against women is a multifaceted and general problem [1]. Although it is prohibited in many countries, violence against women is indeed concealed behind cultural customs, social norms, and religious beliefs. Moreover, violence against women is even considered as merit is some conditions. The results of a study in Iran showed that 49.4% of the surveyed women had experienced violence at least once, with emotional, sexual, and physical violence being 44.4%, 18.9% and 16.4%, respectively [3]. According to the global research conducted under WHO monitoring, 1 out of 4 women is exposed to violence and almost 75% of women in the world have experienced at least violence for once [4]. The results of studies performed in Iran have also confirmed the extent of this social health problem [5]. According to a national survey in 18 provinces of Iran, domestic violence against women has been 66% [6]. Violence against women is one of the behaviors that not only harm women directly exposed to it, but also indirectly other individuals who witness or are aware of it. These individuals are not safe from adverse consequences of violence, and their mental and social well-being is threatened accordingly. Because these people do not feel safe in family and society [2]. Domestic violence is one of the leading causes of health deterioration and premature deaths resulting from violence. Previous studies have designated a 50 to 70% increase in central nervous system problems, mental and emotional health, and antisocial personality disorders [6].

Violence against women is one of the behaviors that directly affect violent people and indirectly affect other people in society. As a result, it endangers the mental and social health of people in society [2]. Domestic violence is introduced as one of the causes of the loss of life with health and the early deaths from violence [7]. Some of the women victims of gender-based violence were pregnant. And it is strange that most of this violence has been committed in physical, mental and social forms by the person closest to them, i.e., their spouses and families. Violence increases women's vulnerability to illness and disability, reduces the extent of their participation in the socio-economic development of society, and increases the risk of harm, death, and a range of physical, emotional, and social problems [8]. Moreover, it can trigger or exacerbate many risk factors like smoking, alcohol use, and drug addiction or high-risk sexual behaviors to escape stress resulted from violence and achieving a false and temporary peace of mind [9]. Pregnancy can be a starting point or sometimes an escalation of domestic violence against a pregnant woman for a variety of reasons, such as decreased sex, misconceptions about pregnancy, and abnormal feelings about pregnancy [10]. Moreover, the dual feelings of women in pregnancy, their vulnerability in this period, and the increased economic pressure are among the effective factors in increasing violence during this period. Women’s dual feelings during this time are the result of the joy related to conceiving a child and the anxiety of facing unknown changes in life following pregnancy [11]. Hence, pregnancy is known as a high-risk period for physical and mental abuse [12]. In this regard, around 1.5 million women in the United States have reported physical or sexual violence by their husband or sexual partner, of which 324,000 were pregnant women [13]. In Iran, women are not immune from such violence such that the level of the applied violence against pregnant women is very high and reported to be more than 60% [14]. Husband’s violence during pregnancy has a deep impact on pregnancy and women’s health decisions. Despite providing the best possible prenatal care, it has been one of the most important factors affecting the worst maternal and fetal outcomes and assessing the nature of children [15]. The importance of the issue is attributed to the wide range of problems and disorders associated with physical and emotional health, improper health behaviors, adverse health behaviors such as delays or non-referrals for prenatal care, smoking and drug abuse, functional disorders, and severe consequences such as suicide and maternal mortality compared to pregnant women with no history of violence [16]. Violence can affect women's reproductive health and increase maternal mortality, morbidity, stillbirth, preterm delivery, abnormal genital bleeding, and pelvic inflammatory disease [17]. There is a relationship between family and social support and the incidence of domestic violence in pregnancy. There seems to a more social and family support is accompanied with the lower incidence of domestic violence against pregnant women [15]. In the health team, the emphasis is on family education to solve problems by the family. Training problem-solving methods has enabled couples to do more for themselves. Through acquiring the ability to work actively, constructively, and collaboratively, they obtain the confidence and strength that they can reduce the pressure-making effects of the obstacles and difficulties in their everyday life [18]. Family consultation features also involve the family of the patient. Although the individuals’ problem is not individually considered in family-based counseling, their problem with their family has been broadly investigated. After obtaining brief information, the family counselor starts advising on changing the atmosphere ruling the family. He/she considers the individual and all the family members and kinship networks involved in the incidence and treatment of problem [19]. Domestic violence against women, in addition to human and human rights, is a major health problem. The physical, psychological, and even social consequences of this issue endanger the health of women in the family and society. Moreover, its effects on pregnancy outcomes cannot be neglected due to the high prevalence of domestic violence in the population of Iranian pregnant women. The current study was designed to specify the effect of family-centered counseling on reducing this problem. The results of this study will hopefully play an effective role in reducing violence against the women population and enhancing their health and security.

## Materials and methods

### Study design and setting

The present study is a clinical trial of two groups (intervention, control) by completing the questionnaire before and after the intervention. Subjects were included in this study based on informed consent and having required inclusion criteria.

### Participants

Inclusion criteria included: obtaining a score of 65 to 128 from the Mohseni Tabrizi Violence Questionnaire (moderate to high levels), not receiving formal education and counseling on domestic violence and spending at least one year living with your spouse. The family of pregnant mothers should also be interested in attending counseling sessions. If there were not any inclusion criteria, the individual would be excluded from the study.

### Sampling methods

The sample size calculation was informed by the results of a study conducted by Bagherzadeh et al. [20]. In this study, we used ϭ1^2^ = 35, ϭ2^2^ = 65, and *d* = 32. Considering the first type error of 5% and the test power of 80% and 10% of sample loss, the required sample size was equal to 46 persons in each group. To do this research, two health centers were randomly selected by lottery from the total three health centers in Sahneh city.

### Randomization procedure and randomization allocation concealment

**Fig. 1:**
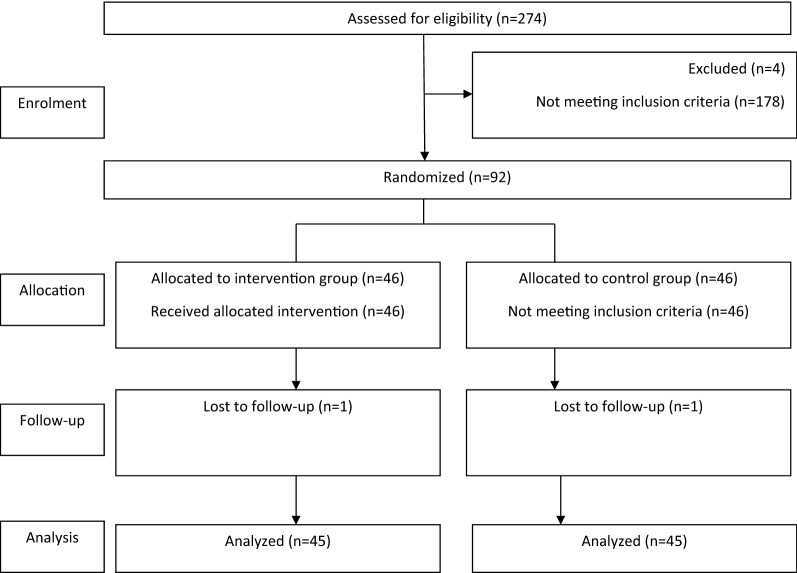
Flow diagram of the participants

Preliminary sampling was performed based on samples available from pregnant mothers visiting the health center. Hamadan was geographically divided into 4 districts after obtaining permission from Hamadan Health Center and the Research Ethics Committee and conducting the necessary coordination. Two health centers were randomly selected from each of north, south, east and west districts using the draws; and 8 health centers were selected. Then, the pregnant women list of these centers was obtained, and allocation sequences were determined using 4th randomized blocking (Fig. 1).The questionnaire was completed for 274 pregnant women who entered the study and expressed their written consent to participate in the study. Sampling was continued until reaching the calculated sample to apply the intervention. Four of the pregnant women under violence were subjected to severe violence according to the scores obtained from the questionnaire. Based on the protocol of pregnant women’s care prepared by the Ministry of Health of Iran, these women were referred to the psychologist as soon as possible in light of the “need to refer”. A total of 178 other pregnant women were subjected to mild violence and thus did not qualify for being included in the study. Next, among 92 selected pregnant women, 89 people were exposed to moderate violence and three people were subjected to severe violence based on the score of the questionnaire. Based on the statistical calculations, 92 pregnant women with inclusion criteria were selected for this study.

### Blinding

Due to the content of counseling sessions, there was no possibility of blindness in this study.

### Instruments

Data collection was conducted using the questionnaire of domestic violence against women of Mohseni Tabrizi et al. prepared and standardized using reliable sources, and synchronized with the social and cultural conditions of Iran [21]. Abolmaali in 2014 used this questionnaire in preparing and constructing a standard questionnaire on domestic violence [22]. The use of this questionnaire in Iran to assess domestic violence has been accepted and used in many studies [23, 24]. The social and cultural circumstances resulting from the Iranian religious norms were assimilated. And in preparing it, attention has been paid to marriage customs in different parts of the country, as well as how couples live after marriage and the type of family. And based on these conditions, it has been localized and psychometric. According to this questionnaire, people who had experienced one of the types of violence by their spouse or one of their family members from the beginning of pregnancy were included in the study.

In this research, the validity of this questionnaire was confirmed by 10 faculty members of Hamadan school of nursing and midwifery and reliability, as well as completing a questionnaire for 20 pregnant women in the study population by Cronbach’s alpha of 0.9. The questionnaire consists of two parts: 1) the demographic characteristics of the pregnant women and their spouses and 2) questions about domains of domestic violence set based on similar studies including physical, psychological, verbal, emotional, financial, and social violence. The domestic violence questionnaire has 31 questions. Questions 1–4 relate to emotional (with questions such as repeated excuses and unfair criticism, expressing tiredness of living together, expressing regret about marriage, paying attention to the weaknesses in the spouse), 5–10 to psychological (with questions such as beating and throwing objects, threatening to divorce and remarry, ignoring sensitivities and desires, provoking jealousy, insulting your friends and relatives, threatening your spouse's loved ones), 11–13 to verbal (with questions such as insults, disrespect, ridicule in the crowd, shouting), 14–18 to financial (with questions such as obsessively controlling family expenses, not giving enough money and money to women, not having access to family income and savings, forcing women to receive their salaries and benefits, forcing women to sell their property), 19–24 to physical (with questions like slapping, pushing, kicking, pulling hair, beating with a belt), 25–28 to sexual (with questions like coercion abortion, unusual sex, coercion, unconventional behavior during sex), and 29–31 (with questions such as opposing your employment, not being allowed to leave the house, being severely monitored, being barred from associating with others and friends and even family members) to the social domain. Each question has four options: never, rarely, sometimes, often, and always, assigned scores of 1 to 5, respectively. A higher score indicates more violence. The sum of scores ranges from 31 to 155. Scores ranging from 31 to 64 fall into the category of mild violence, 65 to 96 in moderate violence, 97 to 128 in high violence, and 129 to 155 very high violence. In this study, individuals with moderate and high levels of violence were selected.

### Intervention

All mothers participating in the study were provided with a complete explanation of the research objectives, the process of their inclusion to the study, counseling meetings, and trusteeship in preserving the information. The participants were free to leave the study at any stage. Pregnant women who were exposed to moderate to high violence according to the questionnaire were referred to a psychiatrist to confirm the violence and then entered the study. Participants in intervention and control groups were visited by a psychiatrist during and after the study to quickly identify any possible complications and risks. If these people had complications, they would be excluded from the study. Pregnant women were asked to choose a member of their family to attend their counseling sessions such as spouse, mother or mother-in-law, etc. And about half of all pregnant women attended counseling sessions with their husbands and the other half with their mothers. Because of the migration of one of the subjects in each group, the study was continued with 45 pregnant women in two groups. The counseling dates were coordinated with the intervention group over the telephone. So, the time for counseling sessions was fixed with the consent of the pregnant woman and her family. In this study, the intervention was conducted at three individual 45-min counseling sessions with a pregnant woman and her family at the health center in the intervention group [25]. On the other hand, the control group, who received only routine prenatal care, was provided with an educational package according to the instructions of the Ministry of Health of Iran at the end of the study. Counseling sessions were conducted according to Greeting, Ask, Tell, Help, Explain, and Refer (GATHER) principles [26]. Also, the control group received only routine prenatal care provided at the health center, at the end of the study, they were provided with a training package. Counseling sessions are conducted by the principal investigator. She is a student of consultation in midwifery (MSc) and works in a health center and is responsible for prenatal care from pregnant women. She has seen the necessary skills courses in this field and has received the relevant certificates. Consulting sessions were conducted according to the principles of GATHER counseling.

The content materials of the counseling were decided according to the information obtained from the questionnaire and the type of violence applied, along with considering the secrecy and safety issues of the family. In the first session, the overall consequences of domestic violence on the outcome of pregnancy were explained to the pregnant mother’s family. The consultation content included the first session on information and explaining the consequences of domestic violence on the pregnancy outcomes for the pregnant mother’s family. The accurate identification of the underlying factors of violence, efforts to eliminate it and empower the family in dealing with life problems, encouraging good communication with each other while improving communication, compliance methods in dealing with problems, the right choice of the alternative behavior for problem-solving, stress management are the proper behavior in marital and sexual relationships. At all sessions, the pregnant woman’s husband and her family were asked to cooperate in better implementation of these recommendations. Then, the next session was determined by the agreement of the pregnant woman.

The researcher took the feedback on previous solutions, investigated its problems in the event of a failure in the recommendations, provided required guidance, and encouraged her husband and family to support the mothers mentally and emotionally.

In the third session, the feedback of the previous sessions was received. During the counseling sessions, the counselor would make phone calls to the participating pregnant women and ask them about the results of the sessions and how their spouses react. To reduce the computational error, the results were based solely on the results of completed questionnaires after the intervention. The content of the counseling sessions, which are based on the principles of GATHER counseling, is given in Box 1.

**Box 1:** Educational content of counseling sessions based on the principles of GATHER

GATHER	Greeting	Greeting and communicating with mother and her family
	Ask	Interacting with mother and asking about her living conditions and stimuli for escalating violence
	Tell	Providing useful information and telling the factors affecting the incidence of violence and ways to prevent it and explaining the consequences of domestic violence on the outcome of pregnancy for the pregnant mother’s family
	Help	Helping the mother and her family choose the best way to decrease the rate of incidence of violence
	Explain	Encouraging good communication with each other and enhancing communication and adaptation methods in dealing with problems and right choosing of the alternative behavior for problem solving, stress Management and improve communication skills, proper behavior in marital and sexual relations. Through all these strategies, the family (spouse) of the woman was asked to help her better observe these recommendations and help her with supporting from her; then the clients gave their Feedback on the information given
	Refer	Pregnant woman’s follow-up was carried out at the next session. The researcher first took feedback on the previous solutions, examined their problems, and then advised them if they did not succeed

Finally, 4 weeks after the end of the sessions, the researcher again completed the domestic violence questionnaire for pregnancy in both groups and analyzed the obtained data.

### Data analyses

After collecting data, they were analyzed using descriptive statistics including tables and numerical indexes. The collected data followed a normal distribution. Then, the t-student test of two independent groups was applied to compare the characteristics of the two groups, and a paired t-test was used to compare them before and after the intervention. The data were analyzed by SPSS16 software and the significance level of the tests was considered 5%.

## Results

### Comparison of demographic characteristics between the two groups

#### Impact of intervention

According to the results, in the intervention group, the mean age of pregnant women and those in the control was 27.1 ± 5.7 and 28 ± 5.5 years, respectively. These values for their spouses were 32.3 ± 6.9 and 32.9 ± 5.3, respectively; the average duration of marriage in the intervention and control groups was 5.7 ± 4.7 and 6.5 ± 4.9 years, respectively; the number of girl children in the intervention and control groups was 1.8 ± 0.3 and 1.2 ± 0.4, respectively; and the number of boy children was 1 in the treatment group and 1.1 ± 0.3 in the control group.

The findings of this study revealed no statistical differences between the two groups. Data were normally distributed and the two groups were homogeneous in terms of demographic characteristics (*P* > 0.05) (Table 1).

**Table 1:** Comparison of some demographic characteristics between the two groups

Variables	Intervention group		Control group		*P*-value
	M	SD	M	SD	
Age of pregnant women	27.1	5.7	28	5.5	0.42
Age of husband of pregnant women	32.3	6.9	32.9	5.3	0.68
Marriage duration	5.7	4.7	6.5	4.9	0.45
Number of daughters	1.8	0.3	1.2	0.4	0.59
Number of sons	1	0	1.1	0.3	0.48

There were no statistically significant differences between the two groups when comparing other qualitative demographic characteristics such as job. However, the majority of mothers in the experimental group (69.5%) and control (71.1%) had lower education than university education level. The variable of the spouse’s education was significantly different and the two groups were not homogeneous (*P* < 0.05). ANCOVA test was applied to moderate the effect of a heterogeneous variable in two treatment and control groups.

#### Impact of intervention

Table 2 shows the comparison of the extent of exposure to violence before and after intervention in the treatment group. As deduced from the table, the overall level of violence exposure decreased after the intervention (*P* < 0.001), indicating the effectiveness of the intervention. The level of psychological violence after the intervention (9.38 ± 2.86) compared to before intervention (13.93 ± 3.50), verbal violence after the intervention (5.2 ± 2.14) compared to before intervention (7.87 ± 2.24), financial violence after the intervention (7.89 ± 2.69) compared to before intervention(11.4 ± 4.52), sexual violence after the intervention (4.18 ± 0.49) compared to before the intervention (4.87 ± 1.78), social violence after the intervention (6.73 ± 3.52) compared to before intervention (8.49 ± 3.64) all decreased. Emotional violence after the intervention (12.36 ± 1.75) was not significantly different from before intervention (12.76 ± 2.99) (*P* = 0.354), indicating that the intervention did not affect the incidence of emotional violence (*P* > 0.05).

**Table 2:** Comparison of the mean scores of the domains of domestic violence against pregnant women in the intervention group before and after intervention

Domestic violence domains	Before intervention		After intervention		*P*-value
	M	SD	M	SD	
Emotional	12.76	2.99	12.36	1.75	0.354
Psychological	13.93	3.50	9.38	2.86	< 0.001
Verbal	7.87	2.24	5.20	2.14	< 0.001
Financial	9.22	3.00	7.89	2.69	< 0.001
Physical	11.4	4.52	7.04	1.68	< 0.001
Sexual	4.87	1.78	4.18	0.49	< 0.001
Social	8.49	3.64	6.73	3.52	< 0.001
Overall violence	68.58	9.21	49.56	8.83	< 0.001

Based on the obtained results, the overall level of violence exposure at the beginning and the end of the study was not significantly different in the control group (*P* = 0.05). As deduced from Table 3, there is no significant difference in any of the violence areas before and after the intervention in the control group (*P* > 0.05).

**Table 3:** Comparison of the mean scores of the domains of domestic violence against pregnant women in the control group before and after intervention

Domestic violence domains	Before intervention		After intervention		*P value*
	M	SD	M	SD	
Emotional	12.35	2.73	12.30	2.83	0.74
Psychological	13.61	3.76	13.80	3.74	0.16
Verbal	8.35	2.14	8.24	2.30	0.41
Financial	9.76	3.55	10.02	3.68	0.09
Physical	10.13	3.76	10.07	3.91	0.65
Sexual	5.41	1.75	5.54	1.85	0.20
Social	8.28	4.20	8.46	4.16	0.08
Overall violence	67.89	7.50	66.35	7.76	0.05

The overall violence of pregnant women in the intervention and control group at the time of entering the study (before intervention) shows no significant difference (*P* > 0.05).

Comparing the two groups after the intervention, except for the emotional violence, shows a significant decrease in other areas of violence, leading to a significant decrease in the overall violence at the end of the study (after intervention) in the intervention group compared to the control group (Table 4) (*P* < 0.001).

**Table 4:** Comparison of the average score of domestic violence against pregnant women in the two control and intervention groups before and after intervention

Domains of violence	Intervention		Control		*P*-value*	*P*-value**
	Before	After	Before	After		
	M (SD)	M (SD)	M (SD)	M (SD)		
Emotional	12.76 (2.99)	12.36 (1.75)	12.35 (2.73)	12.30 (2.83)	0.50	0.92
Psychological	13.93 (3.50)	9.38 (2.86)	13.61 (3.76)	13.80 (3.74)	0.67	< 0.001
Verbal	7.87 (2.24)	5.20 (2.14)	8.35 (2.14)	8.24 (2.30)	0.30	< 0.001
Financial	9.22 (3.00)	7.89 (2.69)	9.76 (3.55)	10.02 (3.68)	0.44	< 0.001
Physical	11.4 (4.52)	7.04 (1.68)	10.13 (3.76)	10.07 (3.91)	0.13	< 0.001
Sexual	4.87 (1.78)	4.18 (0.49)	5.41 (1.75)	5.54 (1.85)	0.14	< 0.001
Social	8.49 (3.64)	6.73 (3.52)	8.28 (4.20)	8.46 (4.16)	0.80	0.04
Overall violence	68.58 (9.21)	49.65 (8.83)	67.89 (7.50)	66.35 (7.76)	0.07	< 0.001

## Discussion

Comparison of domestic violence against pregnant women before and after the intervention in the intervention and control group revealed that domestic violence declined after intervention and there was a significant reduction in the score of overall domestic violence and violence areas (except for emotional violence).

Our findings are consistent with the results of Lopaschuk' study, Beygi et al., Mazhari and et al., Taghdisi et al., Alhusen et al., which is shown that women receiving skilled or community based training or communication counseling in the intervention group had a significant decrease in the severity of domestic violence and an increase in their marital adjustment compared to the control group [17, 25, 27–29]. Low awareness of pregnant women, husbands and their families about the causes of violence and ways to deal with it is one of the causes of violence against pregnant women, so interventions that increase the awareness of women and their families will be effective in reducing violence [30]. Studies have also shown that interventions on violence are reasonably effective in related attitudes, behaviors, and skills, and also change people's knowledge [31].

In our study and other similar studies, the interventions have increased the awareness of women and families. Another reason for the usefulness of our intervention is that family-centered counseling approach has been used. Family-based counseling is a kind of support and decision-based approach aiming at identifying the problem and proposing an appropriate solution [32]. Supportive counseling has reduced the rate of domestic violence by affecting the psychological, physical[33] financial, emotional, verbal, and sexual dimensions [34].Educating family members in controlling and preventing diseases and behavioral disorders can also be very helpful, because there is a strong connection between the family and the health status of its members [35]. Therefore, in order to achieve low-cost, easy, sustainable and less harmful treatment, family cooperation should be used instead of individual treatment to have a positive effect and change the lifestyle. The family, as the most basic element of society, can have a high responsibility in the field of proper and appropriate health care of the pregnant women. Therefore, by educating and engaging influential family members, attitudes toward domestic violence against pregnant women can be changed [36].

Important effects of family-centered counseling include: identifying factors affecting violence and promoting the spouse and family member’s awareness of the physiological and psychological changes in pregnancy, recognizing various areas of violence and the adverse effects of violence against women, her neonate and the pregnancy outcome, and providing appropriate strategies to the factors affecting the violence, especially in each couple to reduce violence [37]. Nevertheless, the results of our study are not consistent with those of Kirk et al. (2017). Despite the decrease in the score of domestic violence in pregnant women by intervention, but no statistically significant difference was observed [38]. This may be due to discrepancies in the study population, sample size, type of intervention and questionnaires to assess domestic violence.

### Limitation of the study

One of the limitations of this study was individual differences such as accuracy, learning power, intelligence, personal patience in pregnant women and their families and its effect on how to implement the given strategies, which was somewhat reduced by increasing the counseling sessions according to individual needs. The low number of the sample in this study due to time limit in sampling is one of the limitations of the study, which reduces its generalizability to the whole society.

## Conclusion

The results indicate that family-based counseling in the studied women significantly reduced the amount of domestic violence against them, confirming the effectiveness of family participation in lowering the violence against pregnant women. The use of professional and public family-based counseling in the prenatal care period of pregnant women is recommended. Applying family-centered counseling to pregnant women, especially with the presence of their spouses, is effective and helpful in reducing domestic violence against pregnant women.

## Data Availability

Data set was produced and created during this research. And because of the confidentiality of the information of the participants is not publicly available, however is available from the corresponding author.
